# Cost-Effectiveness of Positive Memory Training (PoMeT) for the Treatment of Depression in Schizophrenia

**DOI:** 10.3390/ijerph191911985

**Published:** 2022-09-22

**Authors:** Judit Simon, Noemi Kiss, Kees Korrelboom, David Kingdon, Til Wykes, Peter Phiri, Mark van der Gaag, M. Fazil Baksh, Craig Steel

**Affiliations:** 1 Department of Health Economics, Center for Public Health, Medical University of Vienna, Kinderspitalgasse 15, 1090 Wien, Austria; 2 Department of Psychiatry, University of Oxford, Warneford Hospital, Oxford OX3 7JX, UK; 3 Department of Anxiety Disorders, PsyQ Parnassia Group, Psychiatric Center, Lijnbaan 4, 2512 VA The Hague, The Netherlands; 4 Department of Medical and Clinical Psychology, Tilburg University, Warandelaan 2, 5037 AB Tilburg, The Netherlands; 5 Department of Psychiatry, Faculty of Medicine, University of Southampton, Highfield, Southampton SO17 1BJ, UK; 6 Department of Psychology, Institute of Psychiatry, Psychology & Neuroscience, London SE5 8AF, UK; 7 South London and Maudsley NHS Foundation Trust, London SE5 8AZ, UK; 8 Department of Psychology, Faculty of Environmental and Life Sciences, University of Southampton, Highfield Campus, Southampton SO17 1BJ, UK; 9 Research & Innovation Department, Tom Rudd Unit, Southern Health NHS Foundation Trust Moorgreen Hospital, Botley Rd, West End, Southampton SO30 3JB, UK; 10 Department of Clinical Psychology, VU University and Amsterdam Public Mental Health Research Institute, Van der Boechorststraat 1, 1081 BT Amsterdam, The Netherlands; 11 Parnassia Psychiatric Institute, Zoutkeetsingel 40, 2512 HN The Hague, The Netherlands; 12 Department of Mathematics and Statistics, University of Reading, Whiteknights, Reading RG6 6AL, UK; 13 School of Psychology and Clinical Language Sciences, University of Reading, Whiteknights, Reading RG6 6AL, UK; 14 Oxford Health NHS Foundation Trust, Oxford OX3 7JX, UK

**Keywords:** cognitive therapy, schizophrenia, economic evaluation, cost-effectiveness, quality of life, capabilities

## Abstract

The Positive Memory Training (PoMeT) trial demonstrated reduced depression symptoms at 3 months for schizophrenia, but its longer-term outcome and cost impacts remain unknown. This study is a within-trial cost-utility analysis with quality-adjusted life years (QALYs) as outcome based on health-related quality of life (HRQoL) measurement and secondary outcome analyses of capability well-being. The incremental cost-effectiveness of PoMeT was compared to Treatment As Usual only (TAU) over 9 months from the ‘health and social’ care and ‘societal’ perspectives. Uncertainty was explored using bootstrapping and sensitivity analyses for cost outliers and outcome methods. HRQoL improvement was observed for both PoMeT and TAU at 3 months, but reached statistical significance and was sustained only for TAU. There was no change in capability well-being and no significant group difference in QALYs gained over 9 months. Mean intervention cost was GBP 823. Compared to TAU, PoMeT had significantly higher mental health care costs (+GBP 1251, 95% CI GBP 185 to GBP 2316) during the trial, but ‘health and social care’ and ‘societal’ cost differences were non-significant. Compared to the before-trial period, psychiatric medication costs increased significantly in both groups. The probability of PoMeT being cost-effective in the given format over 9 months was <30% and decreased further in sensitivity analyses.. Generalizability remains limited since the before-after cost analysis revealed additional treatment effects also in the TAU group that likely diminished the incremental impacts and cost-effectiveness of PoMeT. It is not clear whether an active post-intervention follow-up could result in sustained longer-term effects and improved cost-effectiveness.

## 1. Introduction

Schizophrenia occurs in approximately 1% of the UK population and accounts for 3% of the NHS budget, or approximately GBP 2.4 billion per year in direct health and social care [1,2]. An additional GBP 5.6 billion is spent on indirect costs to society due to lost productivity of patients and carers, and premature mortality. People diagnosed with schizophrenia have a reduced quality of life, with symptoms impacting their personal, social, and occupational lives. Additionally, this group has a high risk of comorbidities, with about 6–75% (25% modal prevalence) also diagnosed with a depressive disorder and around 1 in 20 committing suicide [3]. Since 2014, offering access to interventions for depression has been a key priority for the implementation of treatment in this patient group [4].

Positive Memory Training (PoMeT) for the treatment of depression in schizophrenia is a short, structured cognitive therapy intervention. A previous form of the intervention has been used successfully to treat auditory hallucinations in people with schizophrenia [5]. In this study it was hypothesized that, if effective, PoMeT would not only bring clinical benefits to patients but could also reduce the need for other forms of depression treatment, prevent expensive hospitalizations, and alleviate carer burden. Due to its shorter duration and less intensive training required for those implementing it, PoMeT could be an affordable and easily implementable technique for the treatment of depression in schizophrenia; an option which could also be cost-effective or potentially cost saving to the health care system.

This study aimed to evaluate the health-related quality-of-life (HRQoL) and capability well-being outcomes, the cost impacts, and the cost-effectiveness of PoMeT, in comparison to Treatment As Usual only (TAU) delivered by mental health professionals from within the NHS Trusts as per local protocols over 9 months, based on evidence from the PoMeT trial [6,7]. It is the first economic evaluation of a psychological intervention for depression in patients with schizophrenia. Besides providing complementary QoL, cost and cost-effectiveness evidence, it was expected that the economic analysis may also shed further light on some of the clinical findings from the trial [6].

## 2. Methods

### 2.1. Study Design and Participants

This within-trial economic evaluation was conducted prospectively alongside the PoMeT trial [6]. The PoMeT trial (ISRCTN99485756) was a two-armed, single-blinded RCT conducted in the UK (by the Berkshire Healthcare National Health Service (NHS) Foundation Trust and the Southern Health NHS Foundation Trust) in an outpatient setting, with 100 participants randomized between 2014 and 2016. It was approved by the NHS Research Ethics Committee (Reference 13/SC/0634). The trial protocol, methodological details, and clinical results, have been previously reported elsewhere [6,7]. Schizophrenia was diagnosed based on DSM-V diagnosis of schizophrenia or schizoaffective disorder. Patients were included if they presented with at least a mild level of depression, defined as a Beck Depression Inventory-II score of 14 or greater [8]. Participants had to have no organic impairment, which was considered the primary diagnosis, be able and willing to provide consent, have no learning disability, and a sufficient level of spoken English to engage with the assessments and clinical intervention. PoMeT training was provided over a period of 3 months in 8–12 structured one-to-one sessions. The intervention was delivered by an accredited cognitive behavioral therapist mental health nurse, a counselling psychologist, and a clinical psychologist, either in a clinic or in the participants’ home. Participants were followed up for 6 months after treatment was completed. The economic evaluation was based on 94 participants, 48 in the PoMeT group and 46 in the TAU group. Five participants were excluded due to fully missing health economic data (both outcomes and costs), while one participant had only baseline health economic data following death unrelated to treatment. Health economic data were collected at baseline, 3, 6, and 9 months, with each assessment relating to the previous 3 month period.

### 2.2. Outcome Assessment

The primary outcome of the health economics analysis was the Quality-Adjusted Life Year (QALY). Health-related quality-of-life (HRQoL) was assessed using the standardized, self-reported EQ-5D-5L questionnaire [9]. This generic measure is recommended for health care resource allocation decisions in the UK [10]. The EQ-5D-5L consists of five dimensions: mobility, self-care, usual activities, pain/discomfort, and anxiety/depression, each with five levels: no problems, slight problems, moderate problems, severe problems to extreme problems. The EQ VAS records the patient’s self-rated health on a vertical visual analogue scale from best to worse health. For the QALY calculations, EQ-5D-5L utility values were developed using the UK TTO value set as recommended earlier [11], half-time correction was applied.

Some studies suggest that for some severe mental health disorders, including schizophrenia, the EQ-5D may not be sensitive enough and may be a too narrow measurement of QoL. Instead, the use of broader well-being measures should be considered [12]. In this study, two additional outcome measures based on the capability approach were used: the ICEpop CAPability measure for adults (ICECAP-A); and the OxCAP-MH [13,14]). The capability approach is particularly relevant in mental health as it distinguishes between capabilities (a person’s opportunities to achieve well-being) and the functioning they achieve (the actual outcomes realized by individuals) [13]. The ICECAP-A measures five attributes: attachment, stability, achievement, enjoyment, and autonomy. The set of index values for the UK population were derived using best-worst scaling and ranges from 0 (no capability) to 1 (full capability). The validity of the ICECAP-A has been tested with the general population and some patient samples [13]. It has also been shown to be suitable for assessing outcome in adults with depression [15]. The OxCAP-MH is a multi-dimensional patient-reported outcome measure originally developed and validated for use in mental health outcome research [14,16,17,18]. It has 16 items rated on a scale of 1–5 that yield an index score ranging from 16–80, which is then converted to a standardized final score on a scale 0 (no capability) to 100 (full capability). The items covered by the questionnaire are: daily activities, social networks, losing sleep over worry, enjoying social and recreational activities, having suitable accommodation, feeling safe, likelihood of discrimination and assault, influencing local decisions, freedom of expression, appreciation of nature, respecting and valuing people, friendship and support, self-determination, imagination and creativity, and access to interesting activities.

Missing data in all outcome measures were under 5% and were balanced between the groups, therefore, multiple imputation was deemed unnecessary. Instead, imputation of the few missing values was based on last-value carried forward in the main analysis and then subjected to sensitivity analysis.

### 2.3. Resource Use and Cost Assessment

Resource use data were obtained for four consecutive three-monthly periods (3 months prior to baseline (-M3) to baseline (M0), baseline (M0) to 3 months follow-up (M3), 3 months (M3) to 6 months (M6) follow-up, and 6 months (M6) to 9 months (M9) follow-up) using an amended version of the patient self-completed Client Service Receipt Inventory (CSRI) [19], and therapists’ diaries. Information on all PoMeT treatment related resource use, other health care resource use (including inpatient stays, outpatient visits, community mental health service contacts, primary care contacts, and psychiatric and depression medications), social care resource use, other health care and broader societal impact (including informal care and lost productivity) were collected. Where relevant, face-to-face visits at home and in the clinic, and phone consultations, were recorded separately. Costs were calculated by multiplying resource use information with UK national-level unit cost estimates (GBP) for year 2016 (Supplementary Table S1) [20,21,22,23,24]. Health care costs reflected the NHS payer perspective. The human capital approach was adopted to estimate lost productivity costs [25]. For study participants in employment, absent work days were multiplied by the average daily UK national salary. Informal care was valued based on average UK hourly salary multiplied by the number of hours family and friends spent on supporting participants as a result of their illness. The 3 months cost information collected prior to the trial period was extrapolated to 9 months through multiplying it by three and then comparing it to the 9 months trial period to explore broader care relevant impacts.

There was no randomly missing resource use information. However, due to a data collection error, 17% of the three-monthly resource use data had to be collected retrospectively from electronic patient records. A positive reliability check of the different data collection methods was carried out based on a sample of patients (*n* = 10) who had both self-reported and electronic records-based data available and showed high levels of agreement. The data collection error was equally distributed between the two groups.

### 2.4. Cost-Effectiveness Analysis

The main cost-effectiveness analysis was an incremental cost-utility analysis with results expressed as incremental cost-effectiveness ratios (ICERs) in terms of the difference on costs (GBP) divided by the difference in QALYs between the PoMeT and the TAU groups from a ‘health and social care’ perspective and from a broader ‘societal’ perspective [10]. Ordinary least squares linear multiple regression framework was used for the analysis adjusted for age, sex, treatment group, and baseline values [26]. Given the 9 months time horizon of the analysis, no discounting was applied either to costs or to outcomes. To determine uncertainty and the 95% confidence interval (CI) of the ICERs, non-parametric bootstrapping was used [27]. Following bootstrapping, joint distribution of the mean incremental costs and effects was illustrated on the cost-effectiveness plane, and the probability of PoMeT being cost-effective in comparison to TAU depending on the society’s maximum willingness-to-pay for a QALY gained based on the net benefit approach, was plotted as a Cost-Effectiveness Acceptability Curve (CEAC) [28,29].

### 2.5. Sensitivity Analyses

The effect of cost outliers in each cost category was explored in a sensitivity analysis. Sensitivity analysis was conducted also on the method of missing outcome data imputation using average before and after values at individual patient level instead of last value carried forward. Furthermore, EQ-5D-5L utilities were recalculated using the UK cross-walk value set [30].

### 2.6. Statistical Analyses

All analyses were carried out according to the intention-to-treat principle. Potential differences in patient characteristics were investigated using *t*-test, chi-square test, or Fisher’s exact test, depending on the type of the variable. For comparisons of the before and during trial periods, paired *t*-tests were used. Means with standard deviations (SD) or mean differences with 95% confidence intervals (95% CI) were reported. A *p*-value of < 0.05 was considered statistically significant. All analyses were conducted using the STATA/MP version 14.2 (Stata Statistical Software: release 14.2; StataCorp LP, College Station, TX, USA) and Microsoft Excel 365 ProPlus.

## 3. Results

### 3.1. Participants

There were no statistically significant differences between the two groups in terms of any of the investigated characteristics (Table 1). The majority of participants were men (75%) and the mean age was 43 years. Most had a primary diagnosis of schizophrenia (69%), the remainder had a diagnosis of schizoaffective disorder or psychosis NOS (31%). Almost two-thirds had severe depression (60%), while 40% had mild/moderate depression. There was some imbalance between the groups in terms of their observed mean baseline EQ-5D-5L (PoMeT: 0.657 vs. TAU: 0.597) and EQ VAS (PoMeT: 52 vs. TAU: 48) values, although the differences did not reach statistical significance (Table 1).

### 3.2. Outcome Results

Main HRQoL results (EQ-5D-5L and EQ VAS) showed improvement for both groups at 3 months, however, the initial utility improvement (+0.036 for PoMeT and +0.059 for TAU) reached statistical significance and was sustained at 6 and 9 month follow-ups only in the TAU group in comparison to baseline (Table 2). No significant changes were observed in capability well-being outcomes (ICECAP-A and OxCAP-MH) during the trial (Supplementary Table S2).

### 3.3. Resource Use and Cost Results

Table 3 shows the main cost results for each cost category. Mean PoMeT intervention cost was calculated at GBP 823 (SD: GBP 354) leading to a significantly higher total mental health care cost per participant in the PoMeT group in comparison to the TAU group (+GBP 1251, 95% CI: GBP 185 to GBP 2316). There were no other statistically significant differences in costs between the two groups, although average non-mental health care costs were substantially lower for PoMeT than for TAU during the trial (−GBP 1498, 95% CI: −3557 to 578).

Comparing costs incurred during the trial period with those extrapolated costs that were incurred before the trial period, significant increases were observed in the cost of psychiatric medications in both groups (Table 3). On average, the PoMeT group had GBP 353 lower costs (95% CI: −GBP 566 to −GBP 141) before the trial period in comparison to during the trial period, while the TAU group had GBP 358 lower costs (95% CI: −GBP 688 to −GBP 28) before the trial compared to during the trial period. Assessing the total number of psychiatric medications taken, significant increases could be observed in both groups (Supplementary Table S3). This included an increase in the number of antidepressants taken during the trial period in comparison to baseline for the full cohort (Supplementary Table S4).

### 3.4. Cost-Effectiveness Analysis Results

**Figure 1:**
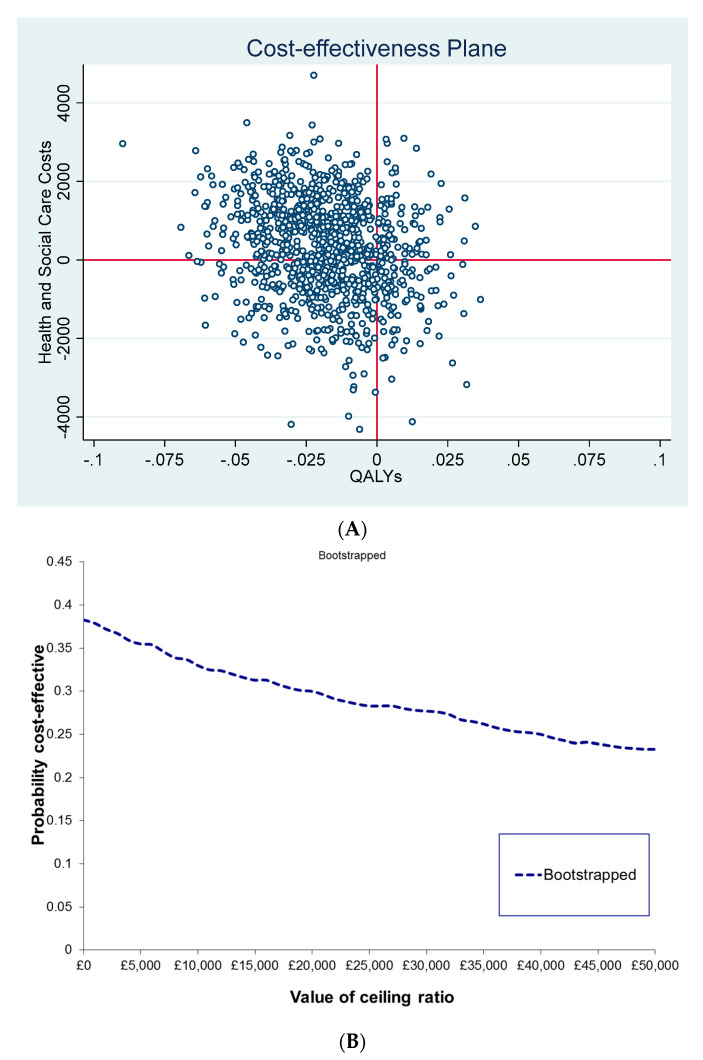
Uncertainty in the cost-effectiveness results (health and social care perspective). (**A**) Cost-Effectiveness Plane: Bootstrapped ICERs (PoMeT vs. TAU); (**B**) Cost-Effectiveness Acceptability Curve (CEAC): Probability of PoMeT being cost-effective in comparison to TAU at different willingness-to-pay thresholds for QALY gained.

Regression adjusted incremental cost, QALY and cost-utility results are reported in Table 4. There were no statistically significant differences between the groups from any of the investigated perspectives. Bootstrapping results illustrated on the cost-effectiveness plane and as CEAC showed great uncertainty around both the outcome and cost differences between the groups with a tendency for PoMeT being more expensive and less effective over 9 months than TAU from the health and social care perspective. Overall, PoMeT had a less than 30% probability of cost-effectiveness at a threshold of GBP 30,000/QALY (Figure 1).

### 3.5. Sensitivity Analyses Results

No difference to the above conclusions was found in the sensitivity analyses where outcome imputation was based on the before and after periods rather than the last value carried forward method, or when EQ-5D-5L utilities were based on the UK cross-walk value set. In the outlier sensitivity analysis, 10 participants were found with cost items that were considered as extreme outliers. All but one outlier cost occurred in the TAU group. For example, one participant in the TAU group frequently visited an occupational therapist with a relevant cost of GBP 3748 over 9 months, and one TAU participant had extreme non-mental health inpatient cost of GBP 39,717, which was more than double that of any other participant. Adjusting for these cost outliers increased the cost difference between the PoMeT and the TAU groups showing PoMeT significantly more expensive from the health and social care perspective, with a tendency of being more expensive also from the societal perspective (Supplementary Table S5). Cost-effectiveness further reduced in both investigated perspectives (Supplementary Table S6) with the probability of PoMeT being cost-effective now close to zero from the health and social care perspective (Supplementary Figures S1 and S2).

## 4. Conclusions

In a previous review, the average minimal clinically important difference for EQ-5D across all diseases was found being 0.18 [31]. Similar to the clinical outcomes, the HRQoL outcome measures did not indicate a longer-term clinically relevant patient improvement by PoMeT over TAU based on evidence from the PoMeT trial. Our finding is in line with recent evidence from a systematic review by Payakachat et al. that showed schizophrenia being one of four conditions where the EQ-5D was non-responsive [32]. Further comparative analysis of the PoMeT outcome data by Helter et al. showed that, although broader capability well-being measures did not indicate clinically relevant improvement by PoMeT either, they correlated better with mental-health specific measures than the EQ-5D-5L for this patient group [33]. Overall, the results of this economic evaluation suggest that in its current format as a short psychological intervention, PoMeT is unlikely to be cost-effective compared to TAU for the management of depression in schizophrenic patients. Sensitivity analyses indicated that these findings are robust to the current methodological assumptions.

On the other hand, the economic evaluation found evidence of increased psychiatric medication taking during the trial period in both groups, indicative of a confounding trial effect in the form of increased treatment also in the TAU group. In addition, the TAU group had on average much higher non-mental health care costs during the study period with some high-cost outliers indicative of intensive alternative service use. These factors potentially explain the reasons behind the sustained average HRQoL improvement observed in the TAU group likely contributing also to the almost GBP 2000 per person reduction in informal care costs in this group, revealed in the before-after analysis. Overall, these trial effects likely diminished the incremental impacts and cost-effectiveness of PoMeT in the current study.

Unexpected clinical improvements have been reported within the control group of a number of trials of psychological therapies for severe mental health problems [34], though such studies typically do not report detailed health economic analyses and, therefore, cannot provide further explorations of potential underlying care-related reasons. It should also be noted that the PoMeT intervention does appear to be associated with a positive effect on depression symptoms at the end of treatment, as indicated by the main clinical analysis [6]. However, treatment gains were not maintained at follow-up. As with other trials, it is, therefore, not clear how the effect and cost-effectiveness of PoMeT would change without the observed trial effects, or whether an active post-intervention follow-up could result in sustained longer-term benefits and improved cost-effectiveness. The optimal number of sessions, timing of therapy with respect to phase of schizophrenia, and with respect to first presentation of schizophrenic symptoms, are all areas where further evidence may be relevant for final treatment decision making.

This study has several limitations. Firstly, it had a relatively small sample size. This poses a problem in that some participants may significantly influence the overall costs and do not allow all cost impacts of the intervention to be detected fully. Although outliers were adjusted for in a sensitivity analysis, a considerable amount of uncertainty remains about these cost impacts, especially for cost categories where only a few participants had actual resource use, or even for outcomes. Another limitation is the necessary use of information from electronic patient records to augment non-randomly missing data on health care resource use. While reliability of this method was tested on a sample for those where information was available from both sources with positive results, the true comparability of these data to those obtained by self-report remains unknown. Furthermore, all cost data refer to year 2016/17. Although this aspect does not impact the validity of the current cost-effectiveness results and conclusions, these costs are not fully reflective of current unit prices. Finally, the study had an overall follow-up of only 9 months that does not allow drawing long-term cost-effectiveness conclusions. Nevertheless, the declining clinical and quality-of-life impacts beyond the initial treatment period suggest that a longer follow-up would have not resulted in different conclusions.

It is important that any conclusions from these findings take into consideration the above limitations as well as the given trial context. The current intervention is based on a specific protocol that targets specific cognitive mechanisms over 3 months. Therefore, the outcome associated with PoMeT is not indicative of those that may be associated with a wider range of therapies, such as generic CBT and third wave cognitive behavioral interventions, such as mindfulness and acceptance-based therapies. Several of these have been included in a recent systematic review of interventions for treating depression and anxiety symptoms in patients with schizophrenia [35]. However, to date, the PoMeT trial is the largest single RCT to evaluate the psychological treatment of depression in schizophrenia.

In conclusion, although the economic evaluation of the PoMeT trial showed an overall negative result, this study shed light on many potential aspects of the PoMeT trial that could influence both the main clinical and the cost-effectiveness results, and would have not been revealed from the clinical analysis alone. It further highlights the importance of considering clinical evidence together with health economic evidence when making health care decisions on new treatment options from trial-based evidence, or deciding on future research priorities and optimizing research designs, especially in the mental health field.

## Figures and Tables

**Table 1:** Health economic analysis cohort characteristics at baseline.

	Health Economic Analysis Cohort				*p*-Value
	PoMeT (*n* = 48)		TAU (*n* = 46)		
	*n*	% or Mean (SD)	*n*	% or Mean (SD)	
**Age**	48	42.92 (9.65)	46	43.57 (11.22)	0.765
**Gender**					0.194
Male	33	68.75	37	80.43	
Female	15	31.25	9	19.57	
**Ethnicity**					0.198
White	44	91.67	41	89.13	
Asian	0	0	3	6.52	
Black	2	4.17	2	4.35	
Other	2	4.17	0	0	
**Primary Diagnosis**					0.571
Schizophrenia	33	68.75	32	69.57	
Schizoaffective or psychosis NOS	15	31.25	14	30.43	
**Age of first psychosis issue**	46	23.09 (9.38)	44	22.05 (9.26)	0.598
**Age of first MH contact**	46	25.17 (8.38)	43	25.49(9.36)	0.868
**Age of first psychosis services**	46	26.87(8.27)	43	26.81 (9.53)	0.977
**Depression Severity**					0.802
Mild/moderate	20	41.67	18	39.13	
Severe	28	58.33	28	60.87	
**Accommodation**					0.249
Own accommodation	7	14.58	8	17.39	
Housing association/local authority accommodation	28	58.33	24	52.17	
In a relative’s/friend’s home	9	18.75	8	17.39	
Residential facilities	4	8.33	6	13.04	
**Living Situation**					0.772
Living alone	26	54.17	25	54.35	
Living with others	22	45.83	21	45.65	
**Higher Education**					0.216
Yes	21	43.75	26	56.52	
No	27	56.25	20	43.48	
**Age left formal education**	47	16.40 (1.30)	45	16.40 (1.54)	0.989
**Employment**					0.554
Employed or self-employed	6	12.50	5	10.87	
Unemployed	41	85.42	40	86.96	
Retired	1	2.08	1	2.17	
**Baseline EQ-5D-5L**	48	0.657 (0.286)	45	0.597 (0.254)	0.301
**Baseline EQ VAS**	48	52 (22.70)	45	48 (18.52)	0.373

PoMeT: PoMeT intervention group, TAU: Treatment As Usual group.

**Table 2:** Health-related quality of life results (EQ-5D-5L, EQ VAS).

	M0 Mean (SD)	M3 Mean (SD)	M6 Mean (SD)	M9 Mean (SD)
**EQ-5D-5L**				
PoMeT (*n* = 48)	0.657 (0.286)	0.693 (0.210)	0.677 (0.256)	0.648 (0.264)
TAU (*n* = 46)	0.600 (0.270)	0.659 * (0.250)	0.671 * (0.247)	0.677 * (0.256)
**EQ VAS**				
PoMeT (*n* = 48)	52 (22.70)	60 * (19.36)	58 (22.98)	53 (24.10)
TAU (*n* = 46)	48 (18.35)	53 (25.33)	51 (23.32)	56 * (22.43)

PoMeT: PoMeT intervention group, TAU: Treatment As Usual group, M: month. * *p* < 0.05.

**Table 3:** Cost results (in GBP, for year 2016/17).

	Before Trial Costs (Extrapolated)			During Trial Costs			Before Trial vs. During Trial Costs	
	PoMeT (*n* = 48)	TAU (*n* = 46)	PoMET vs. TAU	PoMeT (*n* = 48)	TAU (*n* = 46)	PoMeT vs. TAU	PoMeT (*n* = 48)	TAU (*n* = 46)
Cost category	Mean (SD)	Mean (SD)	Diff (95% CI)	Mean (SD)	Mean (SD)	Diff (95% CI)	Diff (95% CI)	Diff (95% CI)
**(A) Mental Health (MH) Community, Outpatient and Inpatient Care**	**2015 (3414)**	**2274 (6093)**	**−259 (−2303 to 1785)**	**1636 (2304)**	**1370 (1798)**	**265 (−580 to 1110)**	**379 (−705 to 1464)**	**904 (−878 to 2685)**
*MH Community Care*	*1253 (2191)*	*1070 (1530)*	*183 (−589 to 955)*	*891 (996)*	*1111 (1743)*	*−220 (−807 to 367)*	*363 (−109 to 835)*	*−41 (−637 to 556)*
Drop-in Center	425 (1339)	208 (632)	217 (−212 to 645)	205 (509)	203 (532)	2 (−211 to 216)	220 (−99 to 539)	6 (−142 to 154)
Community Psychiatrist	0	0	0	5 (23)	5 (14)	−0.2 (−8 to 8)	−5 (−11 to 2)	−5 (−9 to −1)
Community Psychologist	48 (334)	0	48 (−49 to 145)	3 (19)	56 (291)	−53 (−140 to 33)	46 (−52 to 143)	−56 (−142 to 31)
CPN	373 (343)	437 (652)	−63 (279 to 152)	414 (411)	438 (443)	−25 (−200 to 151)	−41 (−181 to 101)	−2 (−193 to 190)
Self Help Group	332 (844)	396 (992)	−64 (−442 to 314)	222 (496)	404 (1442)	−182 (−631 to 268)	110 (−56 to 277)	−8 (−504 to 489)
Drug Alcohol Support	75 (398)	29 (139)	46 (−76 to 168)	43 (234)	6 (33)	37 (−31 to 106)	32 (−16 to 80)	24 (−10 to 58)
*MH Outpatient Care*	*393 (948)*	*353 (399)*	*40 (−257 to 338)*	*263 (266)*	*259 (209)*	*3 (−94 to 101)*	*131 (−160 to 421)*	*94 (−30 to 218)*
Outpatient Psychiatrist	269 (251)	314 (392)	−45 (−181 to 91)	225 (199)	227 (149)	−2 (−74 to 70)	44 (−35 to 122)	87 (−22 to 196)
Outpatient Psychologist	124 (862)	39 (149)	85 (−168 to 339)	37 (177)	32 (151)	5 (−62 to 72)	87 (−170 to 344)	7 (−58 to 71)
*MH Inpatient Care*	*368 (2550)*	*850 (5581)*	*−482 (−2284 to 1319)*	*482 (164)*	*0*	*482 (−146 to 1110)*	*−113 (−1031 to 803)*	*−850 (−807 to 2508)*
**(B) Psychiatric Medication**	**1006 (1011)**	**839 (890)**	**167 (−223 to 556)**	**1359 (1338)**	**1197 (779)**	**162 (−401 to 726)**	−353 (−566 to −141) *	−358 (−688 to −28) *
**(C) Intervention (PoMeT)**	**0**	**0**	**0**	**823 (354)**	**0**	823 (721 to 927) *	−823 (−927 to −721) *	**0**
**MH Care: A + B + C**	**3021 (3795)**	**3113 (6272)**	**−92 (−2234 to 2049)**	**3818 (2803)**	**2567 (2370)**	1251 (185 to 2316) *	**−798 (−1966 to 371)**	**546 (−1291 to 2382)**
**(D) Non-Mental Health Care**	**888 (1729)**	**1112 (2058)**	**−224 (−1005 to 556)**	**758 (1180)**	**2247 (1014)**	**−1489 (−3557 to 578)**	**130 (−305 to 565)**	**−1135 (−3284 to 914)**
*Primary Care*	*182 (220)*	*212 (254)*	*−30 (−127 to 67)*	*175 (175)*	*162 (166)*	*13 (−56 to 83)*	*7 (−32to 45)*	*50 (−22 to 123)*
General Practitioner (GP)	163 (210)	196 (245)	−33 (−127 to 60)	150 (170)	143 (159)	7 (−60 to 75)	12 (−21 to 46)	53 (−17 to 122)
GP Practice Nurse	19 (35)	16 (32)	3 (−11 to 17)	25 (38)	18 (30)	6 (−8 to 20)	−6 (−16 to 5)	−2 (−15 to 10)
*NMH Community Care*	*279 (423)*	*244 (379)*	*−35 (−129 to 199)*	*218 (268)*	*367 (720)*	*−148 (−375 to 78)*	*61 (−58 to 181)*	*−123 (−362 to 117)*
Community District Nurse	11 (62)	7 (50)	4 (−19 to 27)	25 (119)	13 (57)	11 (−26 to 49)	−14 (−33 to 5)	−6 (−29 to 17)
Occupational Therapy	6 (44)	28 (190)	−22 (−79 to 36)	4 (15)	98 (553)	50 (−29 to 129)	2 (−12 to 16)	−70 (−244 to 104)
Physiotherapy	0	10 (65)	−10 (−29 to 10)	9 (45)	22 (77)	−14 (−40 to 13)	−9 (−22 to 5)	−13 (−35 to 10)
Emergency Services	59 (246)	24 (112)	35 (−43 to 114)	20 (79)	77 (185)	−57 (−116 to 2)	39 (−24 to 103)	−53 (−120 to 13)
Alternative Care	25 (171)	0	25 (−25 to 74)	0	9 (58)	−9 (−26 to 9)	25 (−25 to 74)	−9 (−26 to 9)
Other Health Care	178 (302)	176 (234)	3 (−108 to 113)	161 (189)	148 (186)	12 (−64 to 89)	18 (−67 to 103)	28 (−53 to 108)
*NMH Out- and Daypatient Care*	*221 (592)*	*446 (1294)*	*−226 (−643 to 192)*	*164 (328)*	*286 (759)*	*−122 (−365 to 121)*	*57 (−98 to 213)*	*161 (−267 to 589)*
Accident and Emergency	28 (108)	48 (167)	−20 (−78 to 38)	41 (114)	48 (112)	−7 (−53 to 40)	−14 (−61 to 34)	0 (−62 to 62)
NMH Outpatient	130 (319)	399 (1267)	−269 (−655 to 117)	102 (245)	175 (408)	−73 (−212 to 66)	28 (−84 to 139)	224 (−142 to 589)
Daypatient	64 (441)	0	64 (−64 to 192)	21 (123)	63 (367)	−42 (−156 to 72)	43 (−50 to 136)	−63 (−172 to 46)
*NMH Inpatient Care*	*206 (1151)*	*209 (992)*	*−3 (−443 to 436)*	*201 (839)*	*1433 (6379)*	*−1232 (−3141 to 676)*	*5 (−372 to 383)*	*−1224 (−3155 to 708)*
**Health Care: A + B + C + D**	**3909 (3887)**	**4225 (6893)**	**−317 (−2631 to 1998)**	**4576 (2894)**	**4814 (7238)**	**−239 (−2532 to 2055)**	**−668 (−1876 to 541)**	**−589 (−3483 to 2304)**
**(E) Social Care**	**784 (1836)**	**1520 (4282)**	**−736 (−2016 to 633)**	**1118 (2438)**	**745 (2397)**	**373 (−618 to 1363)**	**−335 (−931 to 261)**	**775 (−504 to 2054)**
Social Worker	180 (546)	793 (3686)	−613 (−1718 to 492)	254 (522)	208 (473)	46 (−157 to 250)	−74 (−261 to 112)	585 (−440 to 1610)
Home Helper	103 (698)	78 (519)	25 (−226 to 277)	29 (181)	172 (1058)	−143 (−461 to 175)	74 (−136 to 285)	−94 (−255 to 67)
Housing Worker	4 (22)	35 (110)	−32 (−65 to 1)	31 (178)	21 (97)	10 (−48 to 69)	−28 (−79 to 24)	15 (−13 to 42)
Community Support Worker	478 (1544)	561 (1963)	−83 (−809 to 643)	789 (2180)	334 (1110)	454 (−253 to 1162)	−311 (−1024 to 403)	227 (−385 to 838)
Volunteer Helper	19 (93)	53 (215)	−34 (−103 to 35)	15 (56)	11 (53)	4 (−18 to 27)	4 (−27 to 35)	43 (−17 to 103)
**Health and Social Care: A + B + C + D + E**	**4692 (4324)**	**5746 (7721)**	**−1054 (−3643 to 1535)**	**5694 (3997)**	**5560 (7588)**	**134 (−2378 to 2646)**	**−1002 (−2661 to 257)**	**186 (−2857 to 3229)**
**(F) Total Indirect Costs**	**1617 (3070)**	**4920 (13782)**	**−3303 (−7482 to 876)**	**2300 (4607)**	**2762 (5894)**	**−462 (−2638 to 1713)**	**−683 (−1924 to 558)**	**2158 (−1367 to 5683)**
Lost Productivity (days)	49 (301)	298 (1502)	−250 (−704 to 204)	176 (1027)	95 (457)	81 (−244 to 406)	−127 (−338 to 84)	203 (−236 to 643)
Informal Care	1568 (3081)	4521 (13750)	−3053 (−7224 to 1117)	2124 (4489)	2667 (5908)	−543 (−2702 to 1616)	−556 (−1765 to 653)	1954 (−1547 to 5456)
**Societal: A + B + C + D + E + F**	**6309 (5344)**	**10666 (15826)**	**−4357 (−9283 to 569)**	**7994 (6623)**	**8322 (9843)**	**−328 (−3836 to 3081)**	**−1685 (−3444 to 73)**	**2344 (−2474 to 7161)**

PoMeT: PoMeT intervention group, TAU: Treatment As Usual group, * *p* < 0.05, italics: summary cost sub-categories, bold: summary cost categories.

**Table 4:** Cost-effectiveness results (PoMeT vs. TAU).

Perspective	Cost Difference (95% CI) PoMeT vs. TAU	QALY Difference (95% CI) PoMeT vs. TAU	ICER (95% CI) PoMeT vs. TAU	Interpretation of ICER
**Health and social care**	£270 (−£2191 to £2731)	−0.0177 (−0.0538 to 0.0186)	−£15,254/QALY (−£347,765/QALY to £645,414/QALY)	PoMeT on average is more expensive and less effective
**Societal**	−£458 (−£3783 to £2868)	−0.0175 (−0.0537 to 0.0186)	£26,171/QALY (−£666,926/QALY to £788,883/QALY)	PoMeT on average is less expensive but less effective

PoMeT: PoMeT intervention group, TAU: Treatment As Usual group, QALYs: Quality-Adjusted Life Years, ICER: Incremental Cost-Effectiveness Ratio.

## Data Availability

The data presented in this study are available on request from the corresponding author. The data are not publicly available due to privacy reasons.

## Supplementary Materials

The following supporting information can be downloaded at: https://www.mdpi.com/article/10.3390/ijerph191911985/s1, Figure S1: Uncertainty in the cost-effectiveness results: bootstrapped ICERs (PoMeT vs. TAU) from the ‘health and social care’ perspective adjusted for cost outliers; Figure S2: Cost-Effectiveness Acceptability Curve (CEAC): Probability of PoMeT being cost-effective in comparison to TAU at different willingness-to-pay thresholds for QALY gained from the ‘health and social care’ perspective adjusted for cost outliers; Table S1: Resource use categories and their unit costs (in £, for year 2016); Table S2: Observed capability well-being outcomes (OXCAP-MH, ICECAP-A); Table S3: Number of psychiatric medications taken, by group and observation period; Table S4: Number of antidepressant medications taken, by group and observation period; Table S5: Sensitivity analysis: Cost results adjusted for outliers (in £, for year 2016/17); Table S6: Sensitivity analysis: Cost-effectiveness of PoMeT vs. TAU adjusted for cost outliers.
