# Supplementary material for: Cost-Effectiveness of Positive Memory Training (PoMeT) for the Treatment of Depression in Schizophrenia
Source: Int J Environ Res Public Health. 2022 Sep 22;19(19):11985. doi: 10.3390/ijerph191911985 (PMC9565889; doi:10.3390/ijerph191911985)
Supplement: Supplementary file 1 [file ijerph-19-11985-s001.zip › ijerph-1926801-supplementary.pdf]

# Supplementary material

**Supplementary Table S1:** Resource use categories and their unit costs (in £, for year 2016).

| Resource use | Unit cost (£) | Unit | Source of estimate |
|---|---|---|---|
| **Primary care** | | | |
| GP | 14.60-86.00 | per telephone contact, visit or home visit | [20] |
| Practice nurse (at GP clinic) | 7.90-38.00 | per telephone contact, visit or home visit | [20] |
| **Non-mental health community care** | | | |
| Community district nurse (CDN) | 16.16-106.10 | per telephone contact, visit or home visit | [20] |
| Occupational therapy | 12.47-38.13 | per telephone contact, visit or home visit | [20] |
| Physiotherapy | 23.05-61.18 | per telephone contact, visit or home visit | [20] |
| Emergency service (ambulance) | 7.00-236.00 | per telephone contact, treat & refer, treat & convey | [20] |
| **Mental health community care** | | | |
| Psychiatrist | 17.70-37.57 | per telephone contact, visit or home visit | [21] |
| Psychologist | 61.22-130.00 | per telephone contact, visit or home visit | [21] |
| Psychiatric nurse | 5.20-40.08 | per telephone contact, visit or home visit | [20] |
| PoMeT (intervention) | 86.00 | per contact | [20] |
| Drug/alcohol service worker | 19.50-54.00 | per telephone contact, visit or home visit | [20] |
| Drop-in centre (including street agencies) | 34.00 | per contact | [20] |
| Self-help/support group | 58.40 | per contact | [21] |
| **Mental health outpatient care** | | | |
| Psychiatrist | 49.49-105.08 | per telephone contact or visit | [21] |
| Psychologist | 93.75-199.06 | per telephone contact or visit | [21] |
| **Non-mental health outpatient care** | | | |
| Specific outpatient services | 3.37-473.72 | per service, procedure, test, or contact | [21] |
| **Mental health inpatient care** | | | |
| Psychiatric inpatient general ward | 420.67 | per bed day | [20] |
| **Non-mental health inpatient care** | | | |
| Specific inpatient wards | 423-1468 | per rehabilitation day, per bed day | [22] |
| **Psychiatric medication** | | | |
| Oral medication | Various | per milligram | [23] |
| **Social care** | | | |
| Social worker | 27.52-76.20 | per telephone contact, visit or home visit | [20] |
| Community Support Worker (unqualified) | 13.00-36.00 | per telephone contact, visit or home visit | [20] |
| Home help/home care worker | 4.44-17.33 | per telephone contact, visit or home visit | [20] |
| Housing worker | 7.13-19.74 | per telephone contact, visit or home visit | [24] |
| Voluntary/Charity worker | 7.13-19.74 | per telephone contact, visit or home visit | [24] |
| **Indirect costs** | | | |
| Informal care | 9.21 | per hour | [24] |
| Absenteeism | | | |
| Full time worker | 128.78 | per day | [24] |
| Part time worker | 86.36 | per day | [24] |
| Volunteer worker | 75.56 | per day | [24] |

**Supplementary Table S2.** Observed capability well-being outcomes (OXCAP-MH, ICECAP-A).

| | | **PoMeT** | | **TAU** |
|---|---|---|---|---|
| | *n* | mean (SD) | *n* | mean (SD) |
| **OXCAP-MH** | | | | |
| M0 | 46 | 55 (12.59) | 47 | 56 (13.30) |
| M3 | 45 | 56 (13.65) | 44 | 56 (17.18) |
| M6 | 44 | 55 (15.13) | 44 | 56 (16.09) |
| M9 | 40 | 56 (16.30) | 45 | 58 (16.52) |
| **ICECAP-A** | | | | |
| M0 | 49 | 0.533 (0.210) | 48 | 0.515 (0.214) |
| M3 | 48 | 0.553 (0.198) | 45 | 0.475 (0.284) |
| M6 | 44 | 0.556 (0.228) | 46 | 0.563 (0.258) |
| M9 | 44 | 0.571 (0.207) | 45 | 0.558 (0.243) |

PoMeT: PoMeT Intervention group, TAU: Treatment As Usual group, M: month

**Supplementary Table S3.** Number of psychiatric medications taken, by group and observation period.

| | -M3 to M0<br>mean (SD) | M0 to M3<br>mean (SD) | M3 to M6<br>mean (SD) | M6 to M9<br>mean (SD) | p-value<br>(before vs. during trial periods) |
|---|---|---|---|---|---|
| **PoMeT (*n*= 48)** | 2.50 (1.20) | 2.69 (1.13) | 2.77 (1.19) | 2.85 (1.37) | 0.0115* |
| **TAU (*n*=46)** | 2.36 (1.36) | 2.47 (1.43) | 2.63 (1.42) | 2.67 (1.42) | 0.0013* |

PoMeT: PoMeT Intervention group, TAU: Treatment As Usual group, M: month, *p<0.05

**Supplementary Table S4.** Number of antidepressant medications taken, by group and observation period.

| | -M3 to M0<br>*n* (%) | M0 to M3<br>*n* (%) | M3 to M6<br>*n* (%) | M6 to M9<br>*n* (%) | p-value<br>(before trial vs. during trial periods) |
|---|---|---|---|---|---|
| **PoMeT (*n*=48)** | | | | | |
| **0** | 17 (35%) | 16 (33%) | 16 (33%) | 16 (33%) | |
| **1** | 27 (56%) | 26 (54%) | 26 (54%) | 26 (54%) | |
| **2** | 4 (8%) | 6 (13%) | 6 (13%) | 6 (13%) | |
| **mean (SD)** | 0.73 (0.61) | 0.79 (0.65) | 0.79 (0.65) | 0.79 (0.65) | 0.0832 |
| **TAU (*n*=46)** | | | | | |
| **0** | 22 (48%) | 21 (46%) | 20 (43%) | 19 (41%) | |
| **1** | 17 (37%) | 17 (37%) | 18 (39%) | 19 (41%) | |
| **2** | 7 (15%) | 8 (17%) | 8 (17%) | 8 (17%) | |
| **mean (SD)** | 0.67 (0.73) | 0.72 (0.75) | 0.74 (0.74) | 0.76 (0.74) | 0.0442* |
| **Full cohort (*n*=94)**<br>**mean (SD)** | 0.70 (0.67) | 0.76 (0.70) | 0.77 (0.70) | 0.78 (0.69) | 0.0075* |

M: month, PoMeT: PoMeT intervention group, TAU: Treatment As Usual group, *p<0.05

**Supplementary Table S5.** Sensitivity analysis: Cost results adjusted for outliers (in £, for year 2016/17).

| Cost category | PoMeT (*n*=48) Mean (SD) | TAU (*n*=46) Mean (SD) | PoMeT vs. TAU Diff 95% CI |
|---|---|---|---|
| **(A) Mental Health (MH) Inpatient Community, Outpatient and Inpatient Care** | **1603 (2253)** | **1127 (957)** | **476 (-232 to 1184)** |
| MH Community Care | 858 (854) | 868 (879) | -10 (-365 to 345) |
| Drop-in Center[c] | 205 (509) | 203 (532) | 2 (-211 to 216) |
| Community Psychiatrist | 5 (23) | 5 (14) | -0.2 (-8 to 8) |
| Community Psychologist | 3 (19) | 14 (56) | -11 (-28 to 6) |
| CPN | 414 (411) | 438 (443) | -25 (-200 to 151) |
| Self Help Group | 222 (496) | 202 (464) | 20 (-177 to 216) |
| Drug Alcohol Support | 10 (50) | 6 (33) | 4 (-13 to 22) |
| MH Outpatient Care | 263 (266) | 259 (209) | 3 (-94 to 101) |
| Outpatient Psychiatrist | 225 (199) | 227 (149) | -2 (-74 to 70) |
| Outpatient Psychologist | 37 (177) | 32 (151) | 5 (-62 to 72) |
| MH Inpatient Care | 482 (2164) | 0 | 482 (-146 to 1110) |
| **B) Psychiatric medication** | **1359 (1338)** | **1197 (779)** | **162 (-401 to 726)** |
| **C) Intervention (PoMeT)** | **823 (354)** | **0** | **823 (721 to 927)** |
| **MH Care: A+B+C** | **3785 (2785)** | **2324 (1861)** | **1461 (493 to 2429)*** |
| **D) Non-Mental Health Care** | **758 (1180)** | **811 (1187)** | **-53 (-538 to 432)** |
| Primary Care | 175 (175) | 162 (166) | 13 (-56 to 83) |
| General Practitioner (GP) | 150 (170) | 143 (159) | 7 (-60 to 75) |
| GP Practice Nurse | 25 (38) | 18 (30) | 6 (-8 to 20) |
| NMH Community Care | 218 (268) | 277 (344) | -59 (-186 to 68) |
| Community District Nurse | 25 (119) | 13 (57) | 11 (-26 to 49) |
| Occupational Therapy | 4 (15) | 17 (54) | -12 (-29 to 4) |
| Physiotherapy | 9 (45) | 22 (77) | -14 (-40 to 13) |
| Emergency Services | 20 (79) | 77 (185) | -57 (-116 to 2) |
| Alternative Care | 0 | 0 | 0 |
| Other Health Care | 161 (189) | 148 (186) | 12 (-64 to 89) |
| NMH Out- and Daypatient Care | 163 (328) | 184 (287) | -20 (-146 to 106) |
| Accident and Emergency | 41 (114) | 48 (112) | -7 (-53 to 40) |
| NMH Outpatient | 102 (245) | 127 (248) | -25 (-127 to 76) |
| Daypatient | 21 (123) | 9 (43) | 12 (-26 to 49) |
| NMH Inpatient care | 201 (839) | 188 (758) | 12 (-315 to 340) |
| **Health Care: A+B+C+D** | **4543 (2879)** | **3135 (2061)** | **1408 (378 to 2437)*** |
| **E) Social Care** | 1118 (2438) | 590 (1453) | 528 (-292 to 1348) |
| Social Worker | 254 (522) | 208 (473) | 46 (-157 to 250) |
| Home Helper | 29 (181) | 16 (93) | 13 (-45 to 71) |
| Housing Worker | 31 (178) | 21 (97) | 10 (-48 to 69) |
| Community Support Worker | 789 (2180) | 334 (1110) | 454 (-253 to 1162) |
| Volunteer | 15 (56) | 11 (53) | 4 (-18 to 27) |
| **Health and Social Care: A+B+C+D+E** | **5661 (3980)** | **3726 (2536)** | **1936 (572 to 3300)*** |
| **F) Total Indirect Costs** | **2300 (4607)** | **2762 (5894)** | **-462 (-2638 to 1713)** |
| Lost Productivity (days) | 176 (1027) | 95 (457) | 81 (-244 to 406) |
| Informal Care | 2124 (4489) | 2667 (5908) | -543 (-2702 to 1616) |
| **Societal: A+B+C+D+E+F** | **7961 (6601)** | **6487 (6712)** | **1474 (-1255 to 4202)** |

PoMeT: PoMeT intervention group, TAU: Treatment As Usual group, *p<0.05, **bold: summary cost categories**

**Supplementary Table S6.** Sensitivity analysis: Cost-effectiveness of PoMeT vs. TAU adjusted for cost outliers.

| Perspective | Cost difference (95% CI) PoMeT vs. TAU | QALY difference (95% CI) PoMeT vs. TAU | ICER (95% CI) PoMeT vs. TAU |
|---|---|---|---|
| **Health and social care** | £2,038* (£677 to £3,3399) | -0.0175 (-0.0535 to 0.0184) | -£110,162/QALY (-£900,048/QALY to £818,587/QALY) |
| **Societal** | £1,310 (-£1325 to £3945) | -0.0178 (-0.0537 to 0.0182) | -£74,857/QALY (-£545,977/QALY to £588,983/QALY) |

ICER: Incremental Cost-Effectiveness Ratio, QALYs: Quality-Adjusted Life Years, PoMeT: PoMeT intervention group, TAU: Treatment As Usual group, *p<0.05

## Supplementary Figures

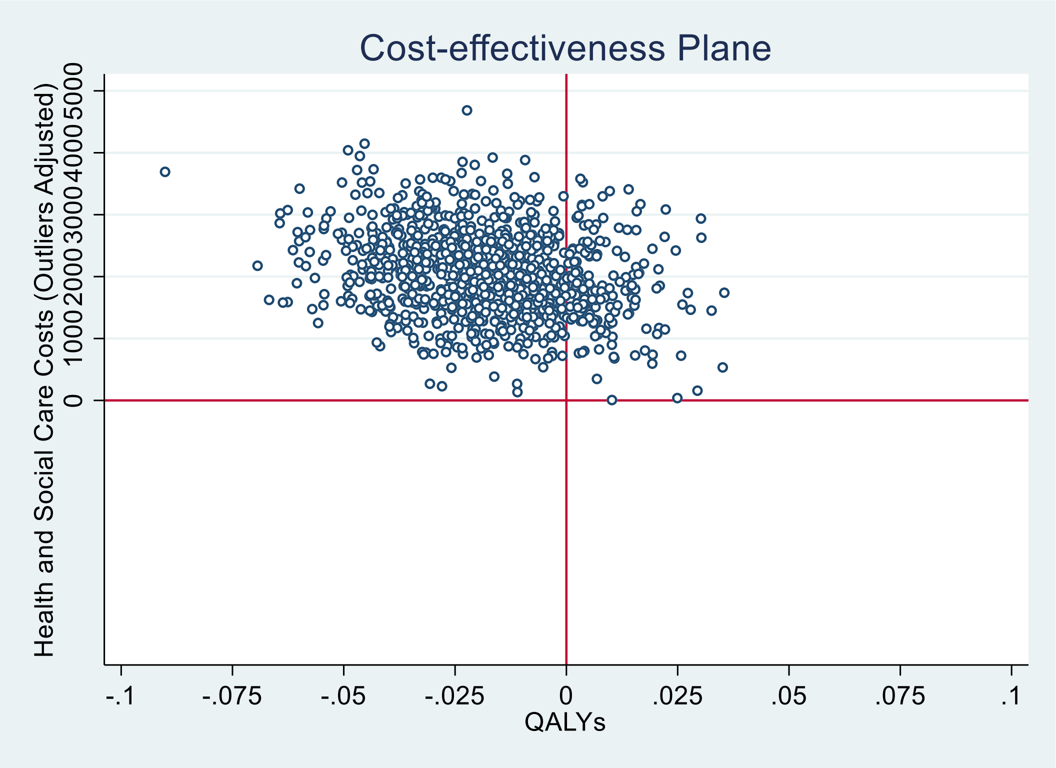


**Supplementary Figure S1.** Uncertainty in the cost-effectiveness results: bootstrapped ICERs (PoMeT vs. TAU) from the 'health and social care' perspective adjusted for cost outliers.

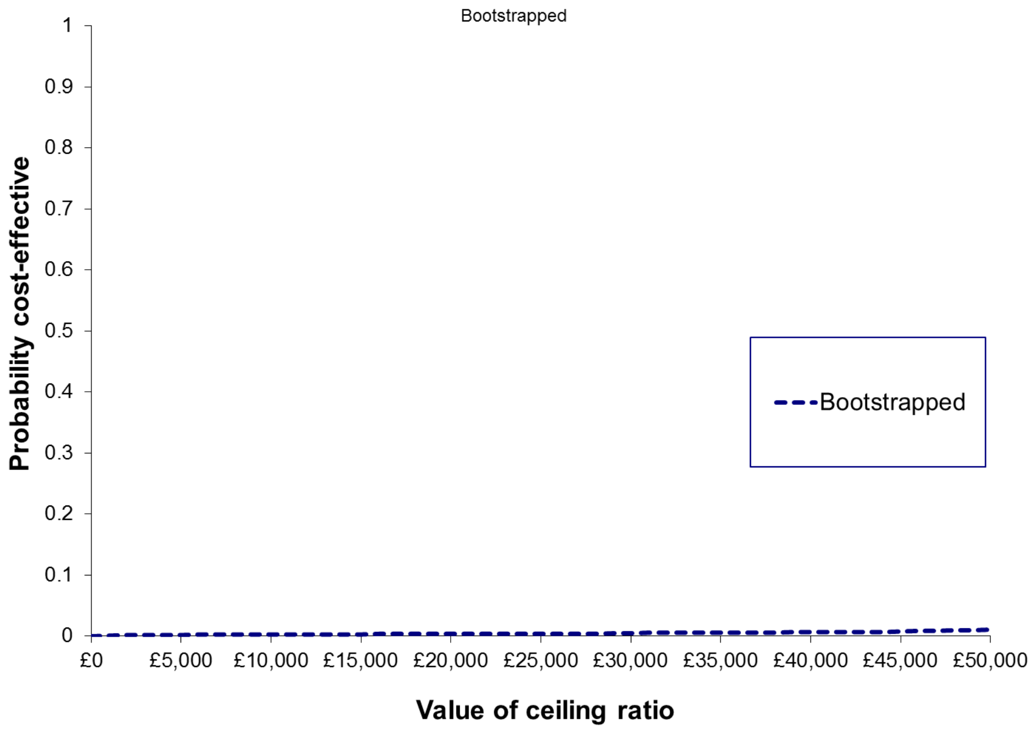


**Supplementary Figure S2.** Cost-Effectiveness Acceptability Curve (CEAC): Probability of PoMeT being cost-effective in comparison to TAU at different willingness-to-pay thresholds for QALY gained from the 'health and social care' perspective adjusted for cost outliers.
